# Assessment of cone-beam CT technical image quality indicators and radiation dose for optimal STL model used in visual surgical planning

**DOI:** 10.1093/dmfr/twae026

**Published:** 2024-06-24

**Authors:** Juha Koivisto, Jan Wolff, Ruben Pauwels, Touko Kaasalainen, Anni Suomalainen, Patricia Stoor, Jani Horelli, Juho Suojanen

**Affiliations:** Department of Physics, University of Helsinki, 00560 Helsinki, Finland; Department of Dentistry and Oral Health, Section of Oral and Maxillofacial Surgery and Oral Pathology, Aarhus University, DK-8000 Aarhus C, Denmark; Department of Dentistry and Oral Health, Aarhus University, DK-8000 Aarhus C, Denmark; HUS Diagnostic Center, Radiology, University of Helsinki, Helsinki, P.O. BOX 224, FI-00029, Finland; HUS Diagnostic Center, Radiology, University of Helsinki, Helsinki, P.O. BOX 224, FI-00029, Finland; Helsinki University Hospital, Helsinki, P.O. Box 63 00014, Finland; Helsinki University Hospital, Helsinki, P.O. Box 63 00014, Finland; Department of Oral and Maxillofacial Diseases, Head and Neck Center, University of Helsinki, P.O. BOX 41, FI-00014, Finland; Planmeca Oy, 00880 Helsinki, Finland; Helsinki University Hospital, Helsinki, P.O. Box 63 00014, Finland; Department of Oral and Maxillofacial Surgery, Päijät-Häme Joint Authority for Health and Wellbeing, Lahti, P.O. BOX 202, FIN-15101, Finland; Cleft Palate and Craniofacial Centre, Department of Plastic Surgery, University of Helsinki, Helsinki, P.O. BOX 281 FI-00029, Finland; Faculty of Medicine, Clinicum, University of Helsinki, P.O. BOX 63, FI-00014, Finland

**Keywords:** cone-beam computed tomography, orbit, Standard Tessellation Language, 3-dimensional

## Abstract

**Objectives:**

The aim of this study was to identify cone-beam computed tomography (CBCT) protocols that offer an optimal balance between effective dose (ED) and 3D model for orthognathic virtual surgery planning, using CT as a reference, and to assess whether such protocols can be defined based on technical image quality metrics.

**Methods:**

Eleven CBCT (VISO G7, Planmeca Oy, Helsinki, Finland) scan protocols were selected out of 32 candidate protocols, based on ED and technical image quality measurements. Next, an anthropomorphic RANDO SK150 phantom was scanned using these 11 CBCT protocols and 2 CT scanners for bone quantity assessments. The resulting DICOM (Digital Imaging and Communications in Medicine) files were converted into Standard Tessellation Language (STL) models that were used for bone volume and area measurements in the predefined orbital region to assess the validity of each CBCT protocol for virtual surgical planning.

**Results:**

The highest CBCT bone volume and area of the STL models were obtained using normal dose protocol (F2) and ultra-low dose protocol (J13), which resulted in 48% and 96% of the mean STL bone volume and 48% and 95% of the bone area measured on CT scanners, respectively.

**Conclusions:**

The normal dose CBCT protocol “F2” offered optimal bone area and volume balance for STL. The optimal CBCT protocol can be defined using contrast-to-noise ratio and modulation transfer function values that were similar to those of the reference CT scanners’. CBCT scanners with selected protocols can offer a viable alternative to CT scanners for acquiring STL models for virtual surgical planning at a lower effective dose.

## Introduction

Over the past decades, patient-specific implants involving 3-dimensional (3D) printed models, drill, and saw guides have been increasingly used for maxillofacial surgical planning and intraoperative guidance.^1^ This has subsequently led to the rapid development of novel image acquisition, data processing, and virtual surgical planning (VSP) methods.^2^ Currently, VSP is mostly based on computed tomography (CT) data.^2^ However, CT scanners are generally associated with a relatively high radiation dose and cost. As VSP is becoming increasingly popular,^3^^,^^4^ alternative imaging modalities such as cone-beam computed tomography (CBCT) have gained interested in order to reduce dose and costs.^5^^,^^6^

To date, most VSP software packages used to virtually plan surgeries require the segmentation of CT/CBCT-generated DICOM (Digital Imaging and Communications in Medicine) sets into Standard Tessellation Language (STL) files typically through manual or automatic thresholding.^7^ However, it must be noted that the DICOM-to-STL conversion of CBCT data sets presents several challenges, such as high noise,^8^^,^^9^^,^^10^^,^^11^ grey value instability,^12^^,^^13^ and the blurring of edges, especially at larger voxel sizes.^14^ One must be aware of image quality bottlenecks, and mitigate these as much as possible through a careful selection of exposure parameters that provide an optimal balance between radiation dose and image quality.

To assess the “quality” of a CT or CBCT scan and the effect of the choice of scan parameters, technical image quality metrics such as the modulation transfer function (MTF) and contrast-to-noise ratio (CNR) can be used. While such metrics are connected to diagnostic image quality,^15–17^ it must be noted that protocols aimed for diagnostic purposes may differ markedly from those required to generate optimal STL models.^18^^,^^19^ Furthermore, in practice, optimizing CBCT protocols for specific tasks can be a cumbersome process, especially outside of a research environment. Whereas medical physicists can assist in setting up and revising scan protocols using metrics such as MTF and CNR, the final choice for these scan protocols is often the result of a subjective decision.

To date, extensive investigations have explored how exposure parameters, head positioning, and segmentation methods influence the accuracy of STL models.^20–24^ However, to the best of our knowledge, the impact of CNR and MTF on the bone volume and area of STL models has not been previously investigated. The objectives of this study were to identify CBCT protocols that offer an optimal balance between effective dose (ED) and bone volume and area depiction, using CT as a clinical reference, and to assess whether such protocols can be defined based on their CNR and MTF.

## Methods

### X-ray scanners

Image acquisitions were performed using a Planmeca VISO G7 CBCT scanner (Planmeca Oy, Helsinki, Finland) with a Superior SXR 130-10-0.5 SC X-ray tube (Superior X-ray Tube Co., Illinois, USA) that has a tungsten (W) target, 10° anode angle, 2.5 mm Al + 0.5 mm Cu filtration and 0.5 mm nominal focal spot size. Furthermore, CT images were acquired using 2 CT scanners; CT1: GE BrightSpeed 16-slice (GE Healthcare Technologies, Waukesha, WI, United States) and CT2: GE Revolution 256-slice (GE Healthcare Technologies, Waukesha, WI, United States). The aforementioned CT scanners are routinely used for VSP and served as the clinical reference for the bone volume and area assessments.^24–27^

In total, 32 different CBCT protocols were investigated, resulting from the combination of normal and ultra-low dose (ULD) dose protocols, symmetric/offset scan modes, 90/100 kV tube voltages, and 4 voxel sizes. The ULD protocols use Planmeca’s Adaptive Image Noise Optimizer (AINO) digital noise reduction filter.^28^ Symmetric scan JAW protocols were designated as J1-J16 (Table 1), and offset scan FACE protocols were designated as F1-F16 (Table 2). A constant field of view (FOV) of 160 mm ×160 mm was selected to encompass the anatomical region commonly used for VSP.

**Table 1.** Exposure parameters of CBCT JAW normal dose protocols (J1-J8). ULD protocols (J9-J16), CNR, MTF (10%) and effective doses.

Protocol	J1	J2	J3	J4	J5	J6	J7	J8
Scan mode	JAW	JAW	JAW	JAW	JAW	JAW	JAW	JAW
Dose mode	Norm.	Norm.	Norm.	Norm.	Norm.	Norm.	Norm.	Norm.
Voxel size (µm)	150	300	450	600	150	300	450	600
Tube voltage (kVp)	90	90	90	90	100	100	100	100
Tube current (mA)	14	14	14	14	12	12	12	12
Exposure time (s)	7.9	4.5	3.6	2.9	5.0	3.2	2.6	2.0
*Q* (mAs)	110	63	50	40	60	38	32	25
DAP (mGy·cm^2^)	1647	931	747	592	1286	827	663	518

CNR	12.8	9.6	16.6	26.9	12.6	9.5	14.4	27.2
MTF 10% (lp/mm)	1.4	1.5	1.1	1.1	1.4	1.4	1.1	1.1
Effective dose (µSv)	209.6	120.0	95.3	76.2	170.6	108.3	90.3	70.5

Protocol	J9	J10	J11	J12	J13	J14	J15	J16
Scan mode	JAW	JAW	JAW	JAW	JAW	JAW	JAW	JAW
Dose mode	ULD	ULD	ULD	ULD	ULD	ULD	ULD	ULD
Voxel size (µm)	150	300	450	600	150	300	450	600
Tube voltage (kVp)	90	90	90	90	100	100	100	100
Tube current (mA)	10	6	10	6	6	4	3	4
Exposure time (s)	2.5	2.5	2.5	1.6	2.5	2.5	2.5	1.6
*Q* (mAs)	25	15	13	10	15	10	8	6
DAP (mGy·cm^2^)	371	237	188	148	330	210	168	131

CNR	15.9	28.6	33.1	39.8	13.8	25.2	28.0	45.3
MTF 10% (lp/mm)	1.4	1.1	1.0	1.0	1.4	1.0	1.0	1.0
Effective dose (µSv)	47.6	29.0	23.8	18.1	43.0	28.2	21.2	17.8

**Table 2.** Exposure parameters of CBCT FACE normal dose protocols (F1-F8). ULD protocols (F9-F16), CNR. MTF (10%) and effective doses.

Protocol	F1	F2	F3	F4	F5	F6	F7	F8
Scan mode	FACE	FACE	FACE	FACE	FACE	FACE	FACE	FACE
Dose mode	Norm.	Norm.	Norm.	Norm.	Norm.	Norm.	Norm.	Norm.
Voxel size (µm)	200	300	450	600	200	300	450	600
Tube voltage (kVp)	90	90	90	90	100	100	100	100
Tube current (mA)	14.0	14.0	14.0	14.0	12.0	12.5	10.0	11
Exposure time (s)	7.9	4.5	3.6	2.9	4.5	3.2	3.2	2.75
Q (mAs)	110	63	50	40	56	40	32	24.75

DAP (mGy·cm^2^)	1146	663	530	424	819	827	663	518
CNR	11.6	6.4	12.0	19.3	6.7	4.2	8.2	13
MTF 10% (lp/mm)	1.5	1.5	1.1	1.1	1.6	1.6	1.1	1
Effective dose (µSv)	155.4	89.0	70.6	56.5	112.5	80.4	64.3	50

Protocol	F9	F10	F11	F12	F13	F14	F15	F16
Scan mode	FACE	FACE	FACE	FACE	FACE	FACE	FACE	FACE
Dose mode	ULD	ULD	ULD	ULD	ULD	ULD	ULD	ULD
Voxel size (µm)	200	300	450	600	200	300	450	600
Tube voltage (kVp)	90	90	90	90	100	100	100	100
Tube current (mA)	8.0	4.5	4.0	4.0	5.0	3.2	3.0	4
Exposure time (s)	3.12	3.12	3.12	2.5	2.5	3.12	2.5	1.575
Q (mAs)	25	14	12.5	10	12.5	10	7.5	6.3

DAP (mGy·cm^2^)	371	237	188	148	271	210	168	518.0
CNR	15.7	15.7	21.3	24.8	9.7	18.0	18.1	12.5
MTF 10% (lp/mm)	1.40	1.11	1.10	1.09	1.27	1.26	1.03	1.2
Effective dose (µSv)	35.3	19.8	17.6	14.1	25.1	20.1	15.1	49.7

### Effective dose assessments

All CT and CBCT ED assessments were performed using an anthropomorphic RANDO RAN102 male head phantom (Radiation Analogue Dosimetry System; The Phantom Laboratory, Salem, NY, United States). The absorbed organ dose measurements were made using a mobile MOSFET device TN-RD-70-W20 comprising 1 TN-RD-38 wireless Bluetooth transceiver, 4 TN-RD-16 reader modules, twenty reinforced high-sensitivity TN-1002RD-H dosimeters, and TH-RD-75M software (Best Medical, Ottawa, ON, Canada).^29^^,^^30^ The equivalent (radiation weighted) dose *H_T_* of the irradiated tissues *T* was calculated using the following equation^31^^,^^32^:

(1) $$H_{T}=w_{R}\sum_{i}fi\cdot \;D_{Ti}$$

where the radiation weighting factor *w_R_* = 1 (Sv/Gy) for X-rays, *f_i_*^33^ is the mass fraction of tissue *T* in phantom layer *I*, and *D_Ti_* is the average absorbed dose of tissue *T* in layer *i*. The summation was performed for all phantom layers.

The ED was calculated by multiplying the equivalent dose for each tissue with the corresponding tissue weighing factors (*w_T_*) according to the International Commission of Radiological Protection (ICRP) guidelines and calculating the sum as follows^33^^,^^34^:

(2) $$E=\sum_{T}w_{T}\cdot \;H_{T},$$

where *w_T_* is the weighting factor of tissue (*T*) and *H_T_* is the equivalent dose in tissue (*T*).

### CNR and MTF measurements

CNR and MTF values were measured on 32 CBCT and 2 CT scans using a QUART DVT_AP phantom along with the user-guided, semi-automatic QUART DVT_TECH software (QUART GmbH, Zorneding, Germany) according to a previous study by Ludlow et al.^33^^,^^34^ The QUART DVT_AP phantom consists of 16-cm diameter cylindrical slabs of Plexiglas with PVC and air elements configured to permit measurements of CNR and MTF-10% based on the DIN6868-161 standard.^35^ Three DICOM slices of each volume were measured, and the results were averaged.

When using the QUART DVT_AP phantom, the CNR is defined as the ratio of the contrast between the PVC-PMMA and the mean image noise of the PVC-PMMA. It describes to what extent a visual contrast can be distinguished from statistical background variations.^36^ The CNR can be described as follows:

$$\mathrm{CNR}=\frac{\left|µF-µB\right|}{\sigma B}$$

where *µF* and *µB* are the mean foreground and background pixel values, respectively, and *σB* is the standard deviation of the background pixel values.^37^

### Selecting candidate protocols for bone volume and area measurements

To justify the use of CBCT scanner for VSP, the CBCT protocols (J1, J2, J5, F1) that exceeded the lowest ED of the CT scanners were excluded from the bone quantity assessments. Furthermore, the remaining protocols were classified into 9 categories based on their CNR and MTF values, with divisions between categories defined based on the lowest CT CNR value (5.5), mean CBCT CNR value (18.3), and mean ± SD MTF value (1.23 + 0.10 lp/mm) (Table 3). Moreover, for categories with a mean or low MTF, protocols having the same CNR-MTF classification were represented by a single protocol from the same category (ie, normal or ULD). It was estimated that segmentations for protocols with low MTF and mean CNR would fail in the orbital region, and these protocols were therefore excluded from the bone assessments. According to the aforementioned criteria, the number of protocols was reduced from 32 to 11 for subsequent bone quantity evaluations.

**Table 3.** Classification of the CBCT and CT protocols based on the CNR and MTF values.

		Low CNR	Mean CNR	High CNR
		(CNR < 5.5)	(5.5 ≤ CNR ≤ 18.3)	(CNR > 18.3)
Low MTF	(MTF < 1.13)	—	J3, J7, F3, F7, F10, F15^a^	J8,^b^ F11^c^
Mean MTF	(1.13 ≤ MTF ≤ 1.33)	—	F8^d^	J6

High MTF	(MTF > 1.33)	F6	J6, J9, J13, F2, F5, F9, CT1, CT2	—

Abbreviations: CNR = Contrast to noise ratio; MTF: Modulation transfer function.

a J3, J7, F3, F7, F10, F15 discarded protocols due to the low MTF-mean CNR combination.

b J8: represented protocols J4, J10, J11, J12, J14, J15, J16.

c F11: represented protocols F4, F12, F16.

d F8: represented protocols F13, F14.

### STL model bone volume and area evaluations

**Figure 1.**
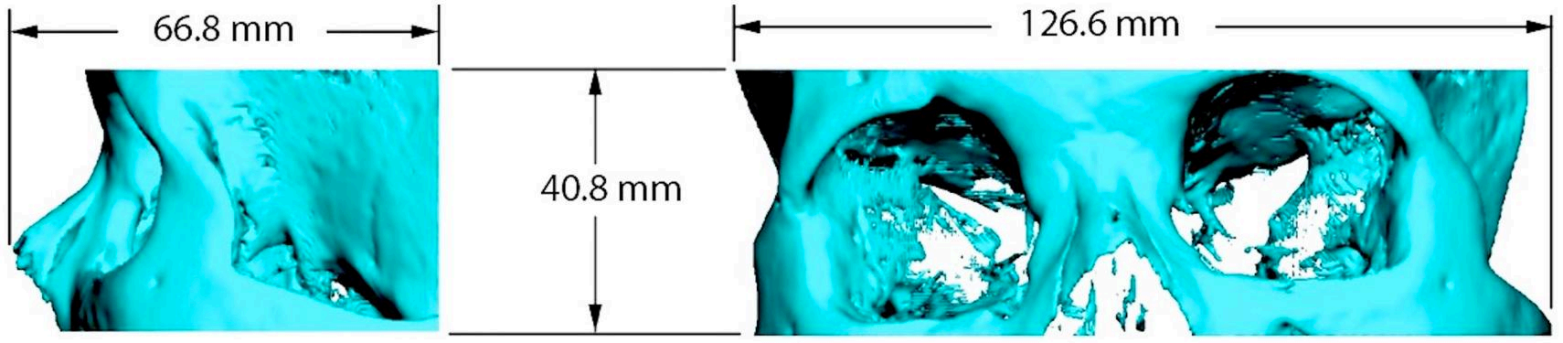
Standard Tessellation Language model dimensions used for bone surface and volume assessments.

The DICOM data sets of the RANDO RAN102 phantom were imported into Romexis (Planmeca, Helsinki, Finland) and were subsequently converted into STL models using global thresholding. The segmentation of all CBCT and CT protocols was performed by an experienced biomechanical engineer (JH) using a standard 1500 grey value threshold for CBCT and a 1230 HU threshold for CT. A scan-specific threshold was not deemed necessary; for the CBCT scanner under investigation, the grey value distribution was consistent among the scan protocols. A standard marching cube algorithm was used to convert the binarized scans into STL models. Next, the bone volumes and areas were measured in a preselected orbital region using a rectangular-shaped region of interest (66.8 × 127.6 × 40.8 mm) (Figure 1). A fully automated superimposing of the STL models prior to the bone area (cm^2^) and bone volume (cm^3^) measurements of all STL models was carried out using 3D Systems Geomagic Freeform 2021 (3D Systems, Rock Hill, SC, United States).^38^^,^^39^

**Figure 2.**
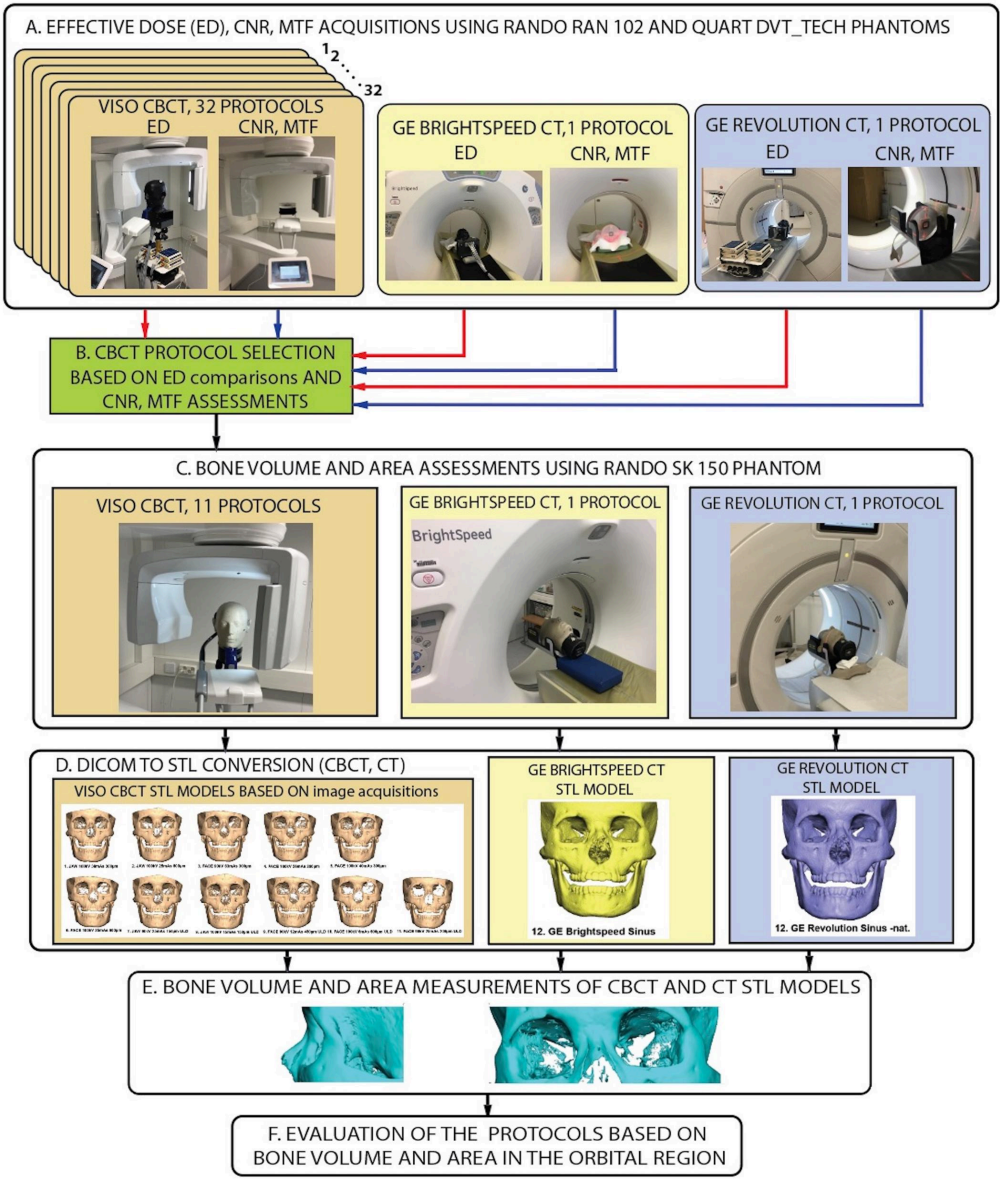
Schematic diagram of the study workflow. (A) Effective dose (ED), contrast-to-noise ratio (CNR)-modulation transfer function (MTF) acquisitions using the cone-beam computed tomography (CBCT) and computed tomography (CT) protocols. (B) CBCT protocol selection based on ED, CNR and MTF assessments. (C) Bone volume and area assessments using an anthropomorphic RANDO SK 150 phantom. (D) (Digital Imaging and Communications in Medicine) DICOM sets into Standard Tessellation Language (STL) files. (E) Bone and volume measurements of CBCT and CT STL models. (F) Evaluation of the protocols based on bone volume and area in the orbital region.

Descriptive statistics were used to express and analyze the results. The absolute bone area and volume of each protocol were calculated. Furthermore, the CBCT-derived bone area and volumes were expressed as a percentage relative to the CBCT mean values (Table 3). A schematic diagram of the study workflow is presented in Figure 2.

## Results

### Effective dose assessments

The EDs calculated for the CBCT protocols ranged between 12.7 µSv (F16: FACE, 100 kV, 6.3 mAs) and 209.6 µSv (FOV 16 × 16 cm, J1: JAW, 90 kV, 110 mAs). The ED for the CT scanners were as follows: GE Revolution: ED = 111.2 ± 8.1µSv, GE Brightspeed: ED = 221 ± 18 µSv.

### Effective dose uncertainty

Type A standard uncertainty of the ED was evaluated in a similar way to that used in a previous study and included all the dosimeters involved in the measurements.^3^^,^^5^ The point dose measurement uncertainty was calculated as the weighted sum of variances and included the statistical measurement error from a previous study measurement error from a previous study, dosimeter and phantom positioning uncertainties (10%, 10%), X-ray source variation (5%), and cable irradiation uncertainties (1%).^11^ The tissue dose uncertainty was dependent on the dosimeter uncertainty and the estimated uncertainty of the fraction irradiated *f_i_* (25%). The tissue dose uncertainties of bone marrow, thyroid gland, oesophagus, skin, bone surface, salivary glands, and brain were 8%, 25%, 24%, 7%, 8%, 6%, and 7%. For the remainder of tissues, the tissue uncertainties for lymphatic nodes, extrathoracic airways, muscles, and oral mucosa were 6%, 11%, 7%, and 6%, respectively. The expanded ED uncertainties (2 SD) were calculated as a weighted sum of the variances of all tissues. The result ranged between 14% and 37% for the CBCT protocols, and between 19% and 26% for MSCT scanners.

### CNR and MTF measurements

**Figure 3.**
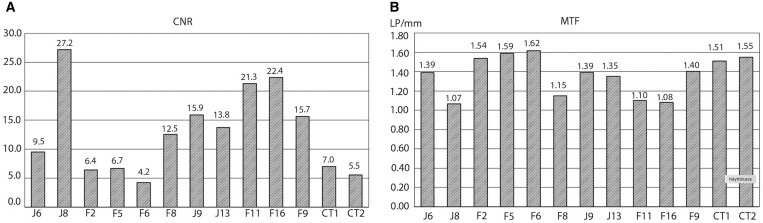
The contrast-to-noise ratio (CNR) (A) and modulation transfer function (MTF) values (B) of 11 cone-beam computed tomography (CBCT) protocols and 2 computed tomography (CT) protocols.

Each volume was measured 3 times, and averages and standard deviations of each parameter were calculated. *CBCT:* The technical image quality assessments for CBCT protocols using the QUART DVT_AP phantom were as follows: CNR = 18.3 ± 4.9, MTF = 1.23 lp/mm ± 0.1. *CT:* The technical image quality assessments for the CT scanners resulted as follows: GE Revolution: CNR = 5.5, MTF = 1.55 lp/mm, GE Brightspeed: CNR = 7.0, MTF = 1.50 lp/mm. For CT, the mean CNR = 6.25 and the mean MTF = 1.525 lp/mm. Each volume was measured 3 times, and averages and standard deviations of each parameter were calculated. The coefficient of variation (ie, standard deviation/average) for CNR and MTF ranged between 1%-10% and 0%-6% for CBCT, and between 1%-7% and 0%-1% for CT, respectively. The EDs, MTF, and CNR values resulting from the CBCT and CT protocols are presented in Tables 1, 2, and 4 and Figure 3.

**Figure 4.**
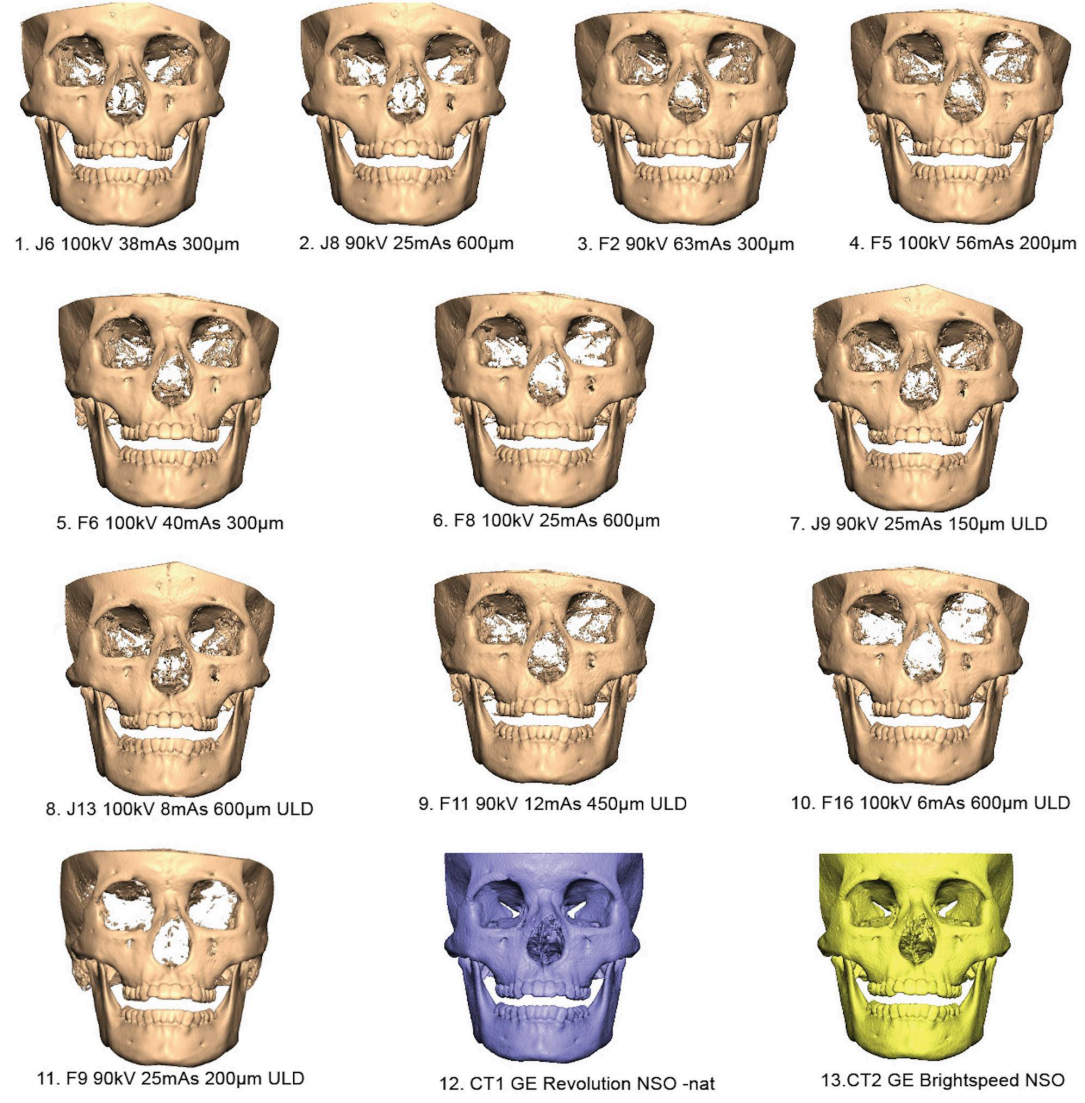
Standard Tessellation Figure 4. models acquired using cone-beam computed tomography (CBCT) (1-11) and computed tomography (CT) protocols (12, 13).

**Table 4.** The exposure parameters, CNR, MTF (10%), effective dose, bone volume, and area of CBCT and MSCT scanners.

Measurement	1	2	3	4	5	6	7	8	9	10	11	12	13

Protocol	J6	J8	F2	F5	F6	F8	J9	J13	F11	F16	F9	CT1	CT2
Device	VISO	VISO	VISO	VISO	VISO	VISO	VISO	VISO	VISO	VISO	VISO	GE	GE
Model	G7	G7	G7	G7	G7	G7	G7	G7	G7	G7	G7	Brightspd	Revol.
Scan mode	JAW	JAW	FACE	FACE	FACE	FACE	JAW	JAW	FACE	FACE	FACE	NSO	NSO-nat

Dose mode	Norm.	Norm.	Norm.	Norm.	Norm.	Norm.	ULD	ULD	ULD	ULD	ULD	—	—
Voxel size (*x*) (µm)	300	600	300	200	300	600	150	150	450	600	200	312.5	312.5
Voxel size (*y*) (µm)	300	600	300	200	300	600	150	150	450	600	200	312.5	312.5
Voxel size (*z*) (µm)	300	600	300	200	300	600	150	150	450	600	200	310	312.5
Tube voltage (kVp)	100	100	90	100	100	100	90	100	90	100	90	120	120
*Q* (mAs)	38.4	25	63	56	40	24.8	25.0	15.2	12.5	6.3	25.0	20.0	15.4

CNR	9.5	27.2	6.4	6.7	4.2	12.5	15.9	13.8	21.3	22.4	15.7	7.0	5.5
MTF 10% (lp/mm)	1.39	1.07	1.54	1.59	1.62	1.15	1.39	1.35	1.10	1.08	1.40	1.50	1.55
Effective dose (µSv)	108.3	70.5	89.0	112.5	80.4	49.7	47.6	43.0	17.6	12.7	35.3	221	111.2
Effective dose ratio^a^	0.97	0.63	0.80	1.01	0.72	0.45	0.43	0.39	0.16	0.11	0.3	1.98	1.00

Bone area (cm^2^)^b^	401.9	329.3	395.4	389.6	386	294.2	386.2	392.3	370.4	284.7	244.8	404.3	417.7
Bone volume (cm^3^)^b^	25.3	27.5	28.1	25.2	25.8	25.9	27.7	28.5	25.3	20.4	15.9	61.0	57,0

∂ bone area %^c^	14	−7	12	11	10	−16	10	11	5	−19	−31	—	—
∂ bone volume %^d^	1	10	12	1	3	3	11	14	1	−19	−37	—	—

Bone area ratio (%) versus CT (average)	98	80	96	95	94	72	94	95	90	69	60	98	102
Bone volume ratio (%) versus CT (average)	43	47	48	43	44	44	47	48	43	35	27	103	97

Abbreviations: CNR = Contrast to noise ratio; MTF: Modulation transfer function.

a Effective dose ratio normalized to GE Revolution NSO-nat protocol.

b Bone area and bone volume in the predefined volume.

c Relative variation percentage from the mean CBCT bone area (352.3 cm^2^).

d Relative variation percentage from the mean CBCT bone volume (25.1 cm^3^).

### CNR and MTF standard deviations

Each volume was measured 3 times, and averages and standard deviations of each parameter were calculated. The coefficient of variation (ie, standard deviation/average) for CNR and MTF ranged between 1%-10% and 0%-6% for CBCT, and between 1%-7% and 0%-1% for CT, respectively.

### Bone volume and area in the orbital region

**Figure 5.**
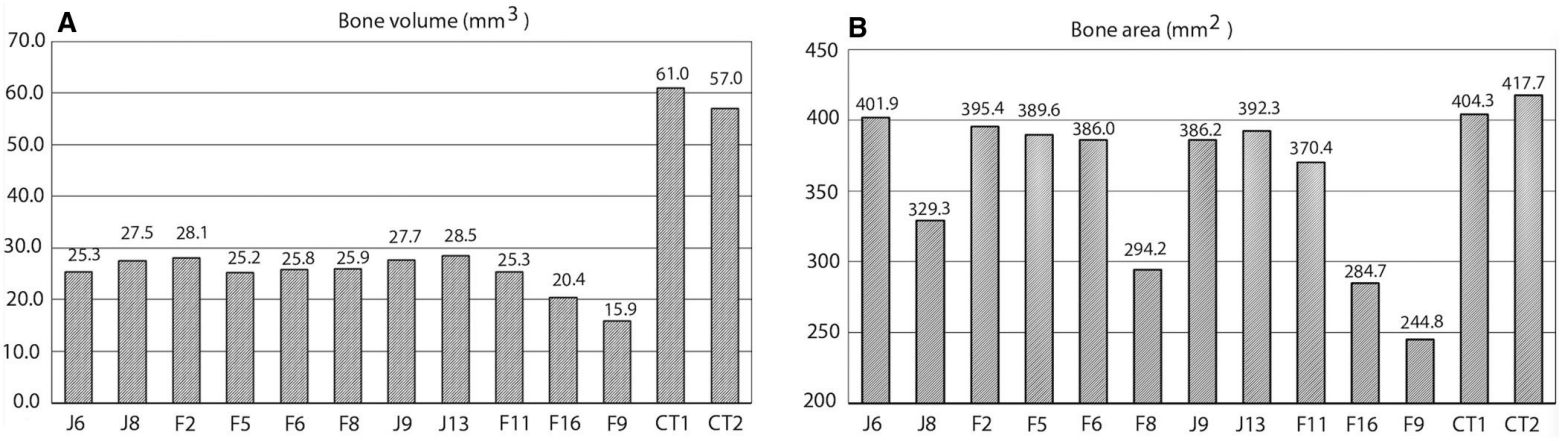
The bone volumes and bone areas of 11 cone-beam computed tomography (CBCT) and 2 computed tomography (CT) protocols.

**Figure 6.**
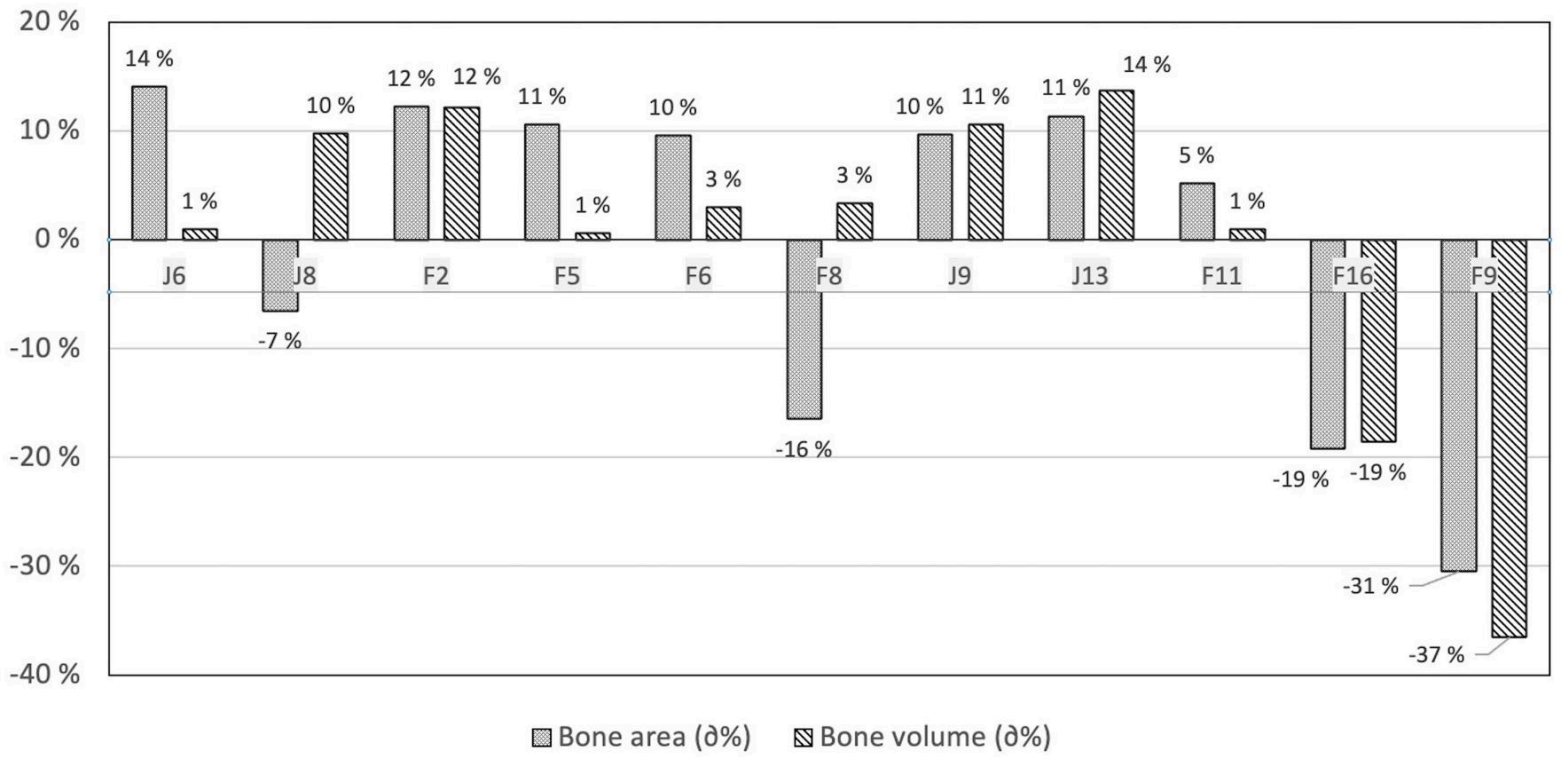
Relative bone volume and bone area variation from the mean values for 11 cone-beam computed tomography scan protocols.

Table 4 shows the bone areas and volumes for the selected CBCT and CT protocols. The bone area for the CT protocols ranged between 404.3 and 417.7 mm^2^ and the bone volume ranged between 57.0 and 61.0 mm^3^. The bone area of the 11 STL models acquired using the CBCT scanner ranged between 244.8 and 401.9 mm^2^. The bone volumes ranged between 15.9 and 28.5 mm^3^. The relative bone area percentage calculated from the mean CBCT value ranged between −31% and +14%. Correspondingly, the relative CBCT bone volume percentage ranged between −37% and +14% of the mean bone volume. The bone volumes (mm^3^) and bone areas (mm^2^) and their relative volume variations from the mean values are presented in Figures 5 and 6.

The highest CBCT bone volume and area of the STL models were obtained using the normal dose protocol F2 (FACE scan mode, 90 kV, 63 mAs, 300 µm voxel size) and the J13 ULD JAW protocol (100 kV, 15.2 mAs, 150 µm voxel size), which resulted in 48% and 96% of the mean STL bone volume and 48% and 95% of the bone area measured on CT scanners, respectively.

### Visual assessment of orbital bone structure

Considerable differences in bone quantities were observed between the CBCT STL and gold standard CT models (Figure 4).

### Assessment of association between CNR and MTF versus bone volume and area

**Figure 7.**
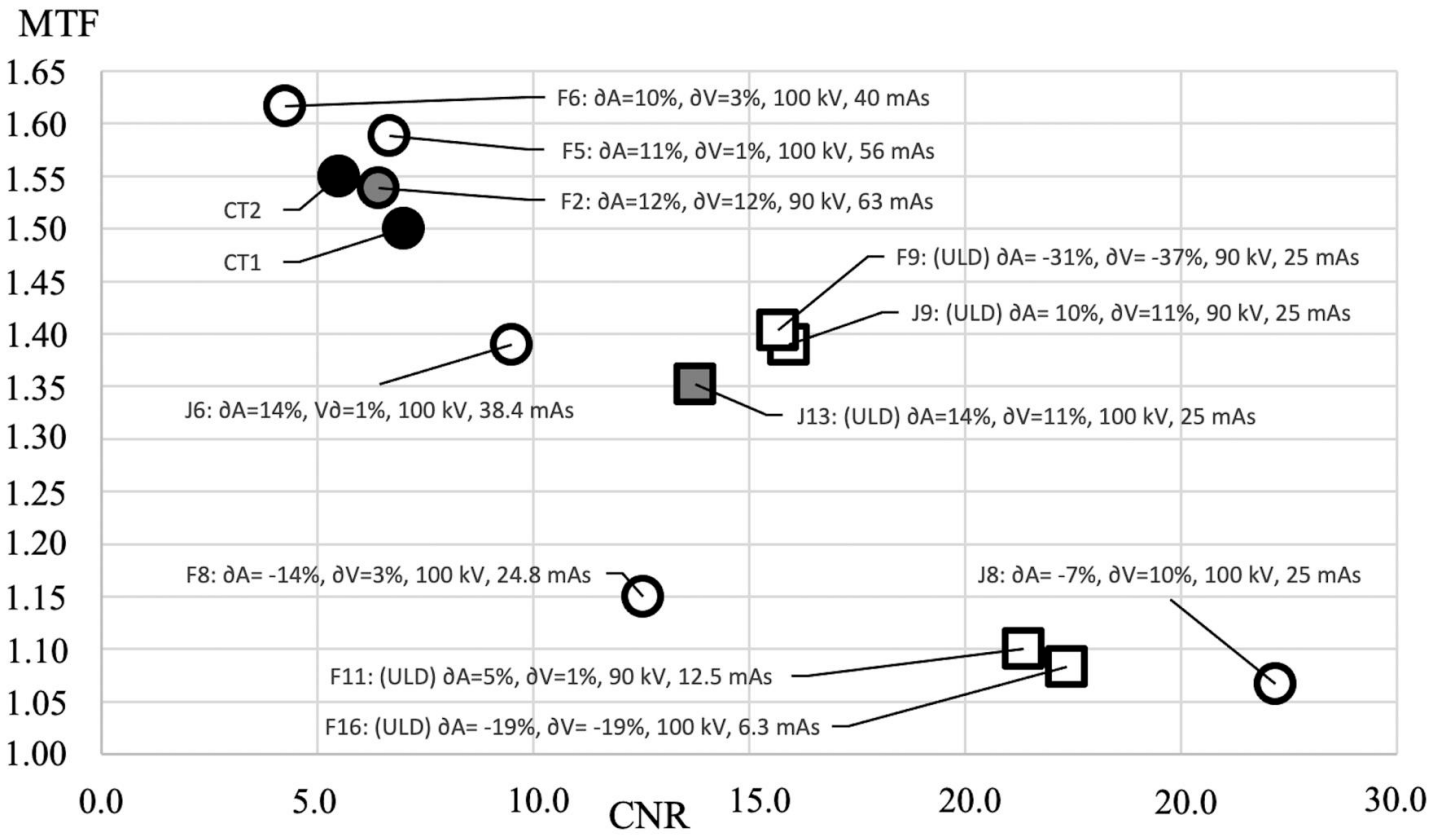
Contrast-to-noise ratio (CNR)-modulation transfer function (MTF) plot of the cone-beam computed tomography (CBCT) and computed tomography (CT) protocols. Solid black dots represent the CT values and the shaded markers represent the CBCT protocols (F2, J13) that demonstrated the largest combination of bone volume and area. The normal dose and ultra-low dose protocols are differentiated using round and square markers, respectively.

In order to depict how the measured CBCT and CT CNR and MTF values correlate with their corresponding bone volumes and areas, their values were plotted in a graph (Figure 7). When comparing CT and CBCT CNR-MTF pairs, it can be seen that the normal dose protocol F2, which produced the largest relative bone volume and surface area, also produced very similar CNR-MTF (CNR = 6.4, MTF = 1.54 lp/mm) as the CT scanners (CNR = 6.25, MTF = 1.525 lp/mm). However, another (ULD) protocol (J13) that uses an AINO digital noise reduction filter offered an STL model with a high relative bone volume and area, despite its CNR-MTF values differing from those of the CT scanners.

## Discussion

The aims of the present study were to assess the performance of STL models acquired using a CBCT scanner for orthognathic surgery planning in the maxillofacial region. This was done by a systematic evaluation of DICOM data acquired using different CBCT protocols and by comparing their EDs, CNRs, MTFs, and bone quantities with those obtained using 2 CT devices commonly used for VSP. The rationale for the study design comes from the need of reducing radiation doses, since the majority of orthognathic surgery patients are adolescents or young adults.

To date, CBCT imaging protocols currently used in clinical settings may often be suboptimal in producing adequate STL models for VSP, especially when high accuracy-requiring anatomical areas are operated or patient-specific surgical guides or implants are used. However, accessibility to conventional CT tends to be more limited as it is predominantly found in hospital environments; for this reason, use of CBCT for VSP imaging is an attempting option. Apart from the inherent limitations of CBCT mentioned above, a reason for poor STL models is likely due to the use of default or improperly selected exposure parameters, which is related to a lack of knowledge and evidence on how these parameters affect the STL model. Furthermore, there has been a misconception that the use of a higher dose would always improve the STL model, resulting in an outcome comparable to that of CT scanners. However, the use of a high patient dose is not considered justified or optimized when a lower dose suffices. This sparked an interest among the authors of this study to investigate the possibilities to identify CBCT protocols that would result in suitable STL models for VSP purposes.

Optimal STL models for VSP should encompass the entire region of interest and offer an accurate, realistic, and detailed depiction of the bony surface and internal structures. However, the segmentation of thin bony structures often fails because of the small voxel size combined with the low CNR resulting from the exposure parameters of the CBCT scanner. In particular, this phenomenon occurs in the orbital region where the STL model is prone to bone loss due to the segmentation process. On the other hand, the thick bony structures surrounding the orbital region cause beam hardening and photon starvation.^40^ In the segmentation process, this can cause bone enlargement of the STL model; thus, the bone volume becomes oversized, but the associated bone area remains disproportionally small.^20^^,^^41^ Commonly, VSP involves reshaping and reconstruction of delicate structures of the orbit that require accurate shape and simultaneous presence of bone volume and area of the STL model. As a result, the performance assessment of the 11 STL models acquired on CBCT protocols was based on their bone volume and area comparisons with the mean bone volume and area values obtained using 2 CT scanners, which were used as the gold standards.

Global thresholding was used in this study; while this represents the most commonly used segmentation method in clinical practice, it does not take local grey value non-uniformities (eg, due to scatter, beam hardening or truncation) into account. Local thresholding or other edge detection methods are less susceptible to this, but often yield suboptimal results barring extensive manual fine-tuning. Recently, novel automated segmentation methods based on deep learning have been proposed; when tailored to specific anatomical structures, these methods could overcome limitations of conventional thresholding.^42^

Interestingly, the normal dose protocol F2 resulted in the largest combination of bone volume and area among the CBCT protocols, while showing similar MTF and CNR values to the CT scanners. This finding is very intriguing as there is currently no straightforward way to translate MTF and CNR values into STL model bone volume and area. However, over the years, CT scanners have been used to produce STL models for VSP. Therefore, it is reasonable to infer that the CNR-MTF pair obtained using CBCT scanners, which are similar to those acquired on using the CT scanners, would also produce an optimal STL model for VSP. That being said, protocol J13 showed a high relative bone volume and area, while having a higher CNR and lower MTF. An important distinction between protocols J13 and F2 is that the former is an ULD protocol that uses a digital noise reduction filter (AINO) affecting both the CNR and MTF.^28^ Brüllmann and d'Hoedt^43^ observed that digital smoothening filters can improve the signal-to-noise ratio with an adverse effect on MTF. Further study is needed to investigate the effect of the reconstruction filter and the possible compensation thereof when connecting technical and diagnostic image quality.

It must be noted that the generation of bone volume and surface area in the STL models is a complex process influenced by several parameters such as voxel size, tube voltage, current, and exposure time, as described by Bavendiek et al.^44–46^ In the current study, the large bone volume observed for STL models based on CT acquisitions could be explained by the large voxel size combined with the partial volume effect (PVE), where the voxel contains a mixture of 2 or more different tissues.^47^^,^^48^^,^^49^ Furthermore, due to the spectral variations and inhomogeneities in the CBCT X-ray beam, the scanned regions of the same density can have different threshold HU values depending on their position in the image volume.^2^^,^^50^^,^^51^ This, in turn, poses difficulties in bone segmentation and may lead to an overestimation of bone volume. However, the aforementioned PVE resulting from the large voxel size can present challenges in thresholding thin bony structures, appearing as poorer depiction of trabecular detail, which subsequently results in a loss of bone surface area, as noted by Cai et al.^20^

In previous orbital wall studies, Jones and Evans^52^ and Park and Yong-Kyu^53^ observed wall thicknesses of 0.37 mm and between 0.131 and 0.245 mm, respectively. Their findings offer an indication for the largest applicable voxel size for bone detection in orbital sockets, and thereby the minimum MTF needed for fine structure depiction. The small voxel size, however, may cause a trade-off, since a higher radiation dose is needed to attain the same CNR level as that of protocols with larger voxels. Furthermore, the increased noise of protocols with small voxels can pose segmentation challenges for automatic marching-cube algorithms, which may struggle to differentiate signal from noise. However, in conditions with reasonable noise levels, small voxel sizes allow for segmentation of fine bony structures, which is reflected as an increase in the STL model’s surface area. These findings align well with the study by van Eijnatten et al,^23^ who investigated the impact of voxel size on the accuracy of bone fill factor, which represents the percentage of CBCT-derived projection divided by the gold standard projection obtained using optical scan. In their study, a smaller voxel size (0.15 mm) resulted in a higher measured bone area compared with a larger voxel size (0.3 mm). Similar observations have been previously made by Pauwels et al,^54^ who concluded that an increase in the trabecular bone structure, and consequently, the bone area, is associated with a small voxel size.

Our results should be interpreted with caution due to the specific conditions under which the data were collected. We used a CBCT scanner from a single manufacturer with default protocol settings. As a result, the findings of this study may not be readily applicable to CBCT scanners from other manufacturers. Further research is warranted to investigate the relationship between bone quantity and exposure parameters across CBCT scanners produced by different vendors.

## Conclusions

The CBCT image acquisition parameters and voxel sizes exert a significant influence on CNR, MTF, and the resulting bone volume and area in STL models. The optimal CBCT bone volume and area were observed for a normal dose protocol “F2.” The optimal CBCT protocol can be defined using CNR and MTF values that were similar to the reference CT scanners’ CNR and MTF values. The results of this study suggest that CBCT scanners hold promise as a viable low-dose alternative to CT scanners for acquiring STL models for VSP in the orbital region, offering the advantage of lower effective radiation dose.
